# Role of Bacterial Lipopolysaccharide in Enhancing Host Immune Response to *Candida albicans*

**DOI:** 10.1155/2013/320168

**Published:** 2013-01-21

**Authors:** Helen Rogers, David W. Williams, Gui-Jie Feng, Michael A. O. Lewis, Xiao-Qing Wei

**Affiliations:** ^1^Tissue Engineering and Reparative Dentistry, School of Dentistry, College of Biomedical and Life Sciences, Cardiff University, Heath Park, Cardiff CF14 4XY, UK; ^2^School of Bioscience, College of Biomedical and Life Sciences, Cardiff University, Museum Avenue, Cardiff CF10 3AX, UK

## Abstract

Human infections involving yeast of the genus *Candida* often occur in the presence of bacteria, and, as such, it is important to understand how these bacteria influence innate host immunity towards *Candida*. Dectin-1 is a cell receptor of macrophages for *Candida albicans* recognition. The aim of this study was to examine dectin-1 expression by monocytes after stimulation with bacterial lipopolysaccharide (LPS), followed by heat-killed *C. albicans* (HKC). Freshly isolated human peripheral blood monocytes (PBMCs) and human monocytes cell line (THP-1) cells expressed low levels of dectin-1. Stimulation with LPS and GM-CSF/IL-4 was found to increase dectin-1 expression in both CD14^+^ human PBMC and THP-1 cells. Enhanced dectin-1 expression resulted in increased phagocytosis of *Candida*. When THP-1 cells were challenged only with HKC, detectable levels of IL-23 were not evident. However, challenge by LPS followed by varying concentrations of HKC resulted in increased IL-23 expression by THP-1 cells in HKC dose-dependent manner. Increased expression of IL-17 by PBMC also occurred after stimulation with *Candida* and LPS. In conclusion, bacterial LPS induces an enhanced immune response to *Candida* by immune cells, and this occurs through increasing dectin-1 expression.

## 1. Introduction

Yeast of the genus *Candida* are frequently carried as harmless commensals at body sites such as the skin, gut, oral cavity, and vaginal tract. However, these fungi are opportunistic pathogens of humans and able to cause serious and potentially life-threatening systemic infections in severely immunocompromised individuals [1–3]. Most infections are, however, superficial, affecting the moist mucosal surfaces of the oral cavity and vagina in debilitated individuals. The occurrence of superficial oral candidosis may arise from a multitude of factors including local immune suppression, reduced salivary flow, poor oral hygiene, smoking, denture wearing, hormonal imbalances, and nutritional deficiencies [4–8]. Furthermore, receipt of broad-spectrum antibiotics has also been implicated with subsequent mucosal and systemic candidal infection [9]. 

The mechanism of immune recognition of *Candida* by the host has been the focus of a number of recent studies. Cells of the innate immune system express an array of pattern recognition receptors (PPRs) such as toll-like receptors (TLRs), which are important in the recognition of microorganisms. Host innate immune cells also express C-type lectin receptors (e.g., dectin-1 and dectin-2) which are PPRs that are key for *Candida albicans* recognition [10, 11]. Dectin-1 is a cell surface molecule exhibiting an inducible expression pattern and an ability to bind to *β*-glucan, which is a major carbohydrate component in the cell wall of *C. albicans* [12, 13]. Recognition of *C. albicans* by dectin-1 not only leads to the formation of receptor synapses for *Candida* phagocytosis [14], but also triggers cell signalling for cytokine production [15]. Dectin-1 deficiency in humans has been shown to be associated with the development of chronic *Candida* infection [16]. 

Engagement of dectin-1 by *Candida* initiates cell signalling through spleen tyrosine kinase (Sky) activation and the CARMA1-related adaptor protein, CARD9, then further downstream activation of NFkB activity for cytokine production [17]. The cell signal triggered by dectin-1 also induces IL-10 and IL-2 production in dendritic cells (DCs) [18] as well as cytokines of the IL-12 family [17]. Some of the cytokines produced by DCs promote Th1 and Th17 cell differentiation resulting in protective immune responses towards infecting fungi [19]. A Th17 response has convincingly been shown to be critical in the host immune response to *C. albicans* infection in animal models [3, 20]. The cytokine IL-23, together with TGF*β* and IL-1*β*, promotes Th17 differentiation and the maintenance of higher IL-17 production [21, 22]. 

Apart from cytokine production by macrophages and DCs, the other important feature in innate immunity is phagocytosis. Phagocytosis is the ability of macrophages and DCs to engulf the pathogen after its recognition by appropriate cell receptors [23–26]. *Candida* killing within a phagosome fused with endosomes and lysosome subsequently occurs [27].

Mucosal colonisation of *C. albicans* occurs at a higher incidence in patients receiving long-term or broad-spectrum antibiotic treatment. This may result from the loss of local competition for *Candida* for nutrients and receptor sites, although an influence on the immune system cannot be discounted. Importantly, limited studies have been undertaken with regards to this latter factor. 

In the present study, we have found that human innate immune cells, CD14^+^ human peripheral blood monocytes (PBMCs), and THP-1 human monocytes exhibit inducible expression of dectin-1 for *Candida* phagocytosis and IL-23 production. Also, bacterial LPS enhances *C. albicans* induced IL-17 (a key cytokine in yeast immunity) production in human PBMCs. The results generated in this study demonstrated that bacteria existing at mucosal sites might have a role in assisting host innate immune cells in *Candida* recognition. This is clearly an important consideration for the treatment and management of patients with *Candida* who are in receipt of antibiotic therapy. 

## 2. Material and Methods

### 2.1. Cell Culture and Stimulation

Collection and isolation of human PBMC from healthy volunteers was done following approval by Cardiff University (DENTL 09/18). Human PBMCs were isolated using density gradient centrifugation as recommended by the manufacturer (GE Life Science, UK). Cells were cultured (with or without stimulation) at 37°C in a 5% CO_2_ enriched atmosphere in RPMI1640 medium supplemented with 10% foetal bovine serum (FBS), containing penicillin and streptomycin. Human THP-1 cells were also cultured in RPMI1640 medium with passage every 3-4 days. 

To investigate dectin-1 expression in human monocytes, the cells were stimulated with increasing concentrations of lipopolysaccharide (LPS) extracted from *Escherichia coli* (Sigma Ltd., UK) followed by challenge with heat-killed *Candida* (HKC) or culture negative controls. Cells were then cultured for different time periods before harvesting and analysis for dectin-1 expression and cytokine production.

### 2.2. Preparation of Heat-Killed **C. albicans**

Three well-characterised clinical isolates of *C. albicans* were used for the challenge studies [28]. The isolates had previously been identified based on traditional biochemical analysis as well as sequencing of rDNA gene sequences. The isolates were cultured overnight at 37°C in yeast nitrogen base medium supplemented with glucose. The cells were centrifuged and washed with PBS (×3) before being heated at 98°C for 10 minutes. Yeast viability was then assessed by culture on Sabouraud dextrose agar to confirm total cell death. 

### 2.3. Cell Phagocytosis Assay and FACS Analysis

To examine DC and monocyte-mediated phagocytosis of HKC, the HKC were initially stained with propidium iodide (PI red fluorescent dye). Briefly, 10 *μ*L of 1 mg/mL PI was added to 200 *μ*L of 10^7^ HKC/mL in PBS and incubated for 30 minutes on ice before washing with cold PBS (×3).

To analyse phagocytosis of the HKC by human PBMCs and THP-1 cells, the cells were stimulated overnight with increasing concentrations of LPS followed by addition of 10^5^ HKC which had been stained with PI. This preparation was then cultured for further 2 hours. The cells were then washed (×3) with cold PBS before fixation of the cells with 2% paraformaldehyde in PBS. Cells without addition of *Candida* served as negative controls. Phagocytosis of PI-stained HKC was measured using fluorescent microscopy or FACS analysis. Negative controls were used for setting FACS gating in order to obtain the percentage of cells that had phagocytosed PI-labelled HKC. An increased PI fluorescent signal was evident by a shift towards the right in FL2 histogram plots, which indicated a higher phagocytotic ability of the cells. In some experiments, human PBMCs were stained with fluorescein isothiocyanate (FITC) conjugated anti-dectin-1 (Abcam, USA). The association of dectin-1 expression with HKC phagocytosis was then detected by FACS analysis. 

### 2.4. Detection of Dectin-1^+^ and CD14^+^ Expression in Human PBMCs and THP-1 Cells

To detect dectin-1 expression in human monocytes, with or without LPS stimulation, human PBMCs and THP-1 cells were cultured overnight with increasing concentrations of LPS (0, 10, 100, and 200 ng/mL). Cells expressing dectin-1 were then detected by staining with an anti-dectin-1 specific antibody (Abcam) in combination with anti-human CD14 antibody (ImmunoTools, Germany) and isotype control antibodies. Cells were then washed (×3) with PBS and subsequently fixed in 300 *μ*L of FACS buffer containing 2% paraformaldehyde. Samples were analyzed by collecting 10,000 events using a FACSCalibur flow cytometer (BD Biosciences, UK). CD14^+^ cells in PBMC were gated and increased percentages of dectin-1 positive cells calculated and compared to cells without LPS stimulation.

### 2.5. Real-Time RT-PCR

Total RNA was prepared using the RNeasy Mini Kit with the QIAshredder spin columns (QIAGEN) and “on-column” degradation of genomic DNA with 340 units/mL of RNase-free DNase I (Invitrogen) for 15 min. Total RNA (1 *μ*g) was reverse transcribed into cDNA using Superscript II RNase H reverse transcriptase (200 units, Invitrogen) and 100 ng of random primers in a total volume of 20 *μ*L for 50 min at 42°C following the manufacturer's instructions. Levels of mRNA of dectin-1, IL-12p40, and IL-23p19 were quantified on an ABI PRISM 7000 Sequence Detection System (Applied Biosystems). The following primer pairs were used to determine human dectin-1, IL-12p40, and IL-23p19 mRNA levels in comparison to the human “housekeeping” GAPDH gene: human dectin-1 sense 5′-GCT TAA TTG GAA AGA AGA GAA GA, anti-sense 5′-GAT TAA AGG GAA ACA GGT ATC TT; human IL-12p40 sense 5′-TGA AGA AAG ATG TTT ATG TCG TAG AAT, anti-sense 5′-GGT CCA AGG TCC AGG TGA TA; human IL-23p19 sense 5′-AGC TTC ATG CCT CCC TAC T, anti-sense 5′-AGG CTT GGA ATC TGC TGA G, and human GAPDH sense 5′-TCC CGC TTC GCT CTC TGC TCC TC, and anti-sense 5′-GAC CAG GCG CCC AAT ACG ACC AAA T. To quantify mRNA levels in cells, relative gene expression was determined using a SYBR Green qPCR kit (Bio-Rad, USA), and all samples were run in triplicate. The cycle threshold (CT) value of each sample was determined for calculation of the 2^−ΔΔ^ CT the data were expressed as expression-fold relative to the control. 

### 2.6. IL-23 ELISA

IL-23 levels in cell culture medium were determined by sandwich ELISA (eBiosciences Inc.) following the manufacturer's recommended protocol. Briefly, wells of a high protein binding 96-well microtitre plate (Fisher) were coated overnight at 4°C with 50 *μ*L of monoclonal anti-human IL-23p19p specific antibody in 0.1 M NaHCO_3_, pH 8.5. After blocking nonspecific binding with 10% FBS in PBS for 2 h at 37°C, cleared cell supernatants (50 *μ*L) and human IL-23 recombinant protein standards (5 ng/mL in double dilution) were added to the plate in triplicate. IL-23 protein was captured overnight at 4°C. After thoroughly washing the plate with PBS containing 0.05% Tween 20, specific bound IL-23 was detected with a biotin-conjugated anti-human IL-12p40 antibody (2 h at 37°C), followed by incubation with StreptAvidin-HRP for 2 h at 37°C. Results were visualised by adding 50 *μ*L of SureBlue TMB peroxidase substrate for 15–30 minutes at room temperature, followed by addition of 50 *μ*L of the stop solution. The optical density of each well at 450 nm was determined, and the IL-23 concentration was calculated based on the IL-23 standards.

### 2.7. Statistical Analysis

Results were presented as mean ± standard deviation (SD). All statistical analyses were performed using Minitab software. A Student's *t*-test analysis was also conducted. A *P* value of <0.05 was deemed statistically significant. 

## 3. Results 

### 3.1. Dectin-1 Expression by THP-1 Human Monocytes

To study the dectin-1 expression by human monocytes, we first examined the dectin-1 expression in THP-1 cells. Without LPS stimulation, THP-1 cells did not express high levels of dectin-1 mRNA. LPS stimulation was, however, found to induce a dose-dependent increase in dectin-1 expression detected by both RT-qPCR for mRNA and FACS staining for cell surface protein expression. 

**Figure 1:**
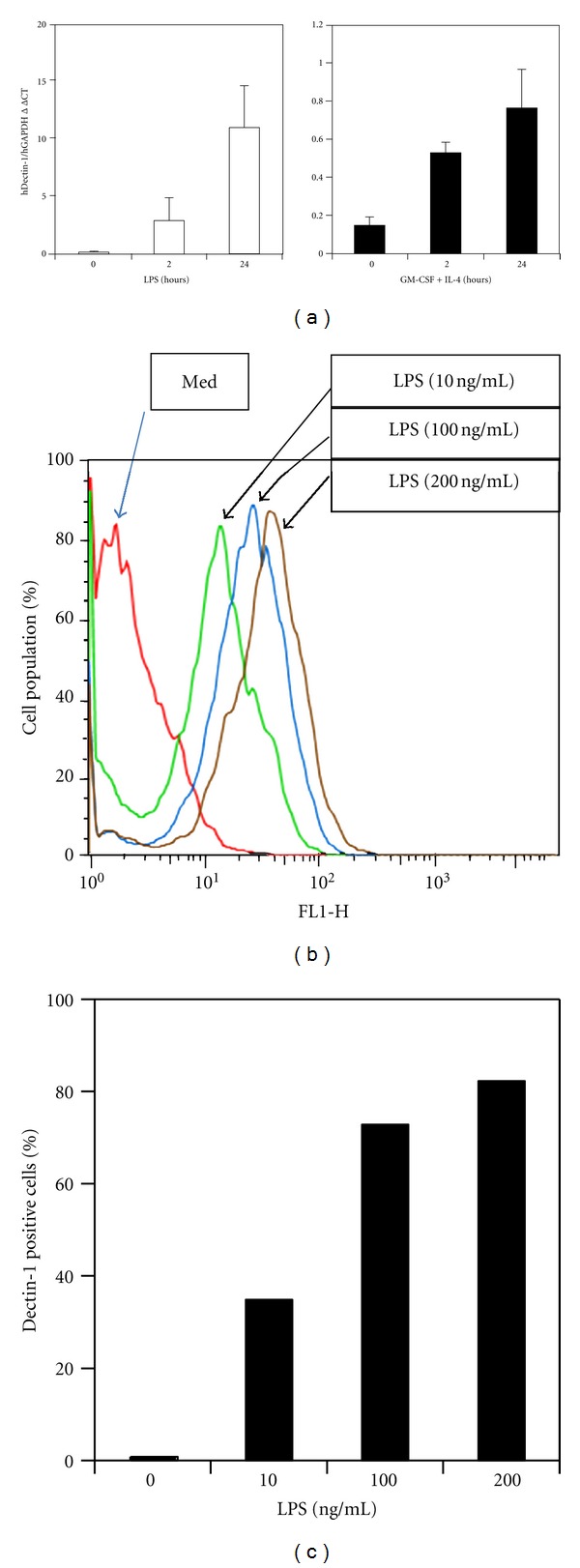
Dectin-1 expression was induced in a human monocyte (THP-1) cell line by LPS. (a) Dectin-1 RT-qPCR demonstrated increased expression of mRNA in THP-1 cells at 2 and 24 h after LPS stimulation. The expression was also increased at the time points with GM-CSF and IL-4. (b) Cell surface dectin-1 was increased with LPS stimulation in a dose-dependent manner. (c) The percentage of cells with higher level of dectin-1 expression by FACS analysis. The results were representative of 2 independent experiments.

GM-CSF and IL-4 are cytokines which normally drive DC maturation from human PBMC [29]. When THP-1 cells were cultured with 20 ng/mL GM-CSF and 10 ng/mL IL-4 (concentrations typically used for DC differentiation), upregulation of dectin-1 was readily detected by mRNA levels after 2 h stimulation, and this was further increased after 24 h (Figure 1(a)). However, the increased levels of dectin-1 mRNA expression were marginal (ΔΔCT = 0.76 ± 0.20) and not comparable to those following LPS stimulation in cultures (ΔΔCT = 10.90 ± 3.61). Indeed, LPS stimulated significant levels of dectin-1 mRNA at both 2 h and 24 h poststimulation (Figure 1(a)). FACS analysis showed an LPS dose-dependent increased dectin-1 expression in THP-1 cells (Figure 1(b)), and quantified results showed a percentage increase in dectin-1 positive THP-1 cells with increasing doses of LPS stimulation (Figure 1(c)).

### 3.2. Dectin-1 Expression and HKC Phagocytosis in CD14^+^ Human PBMC after LPS Stimulation

**Figure 2:**
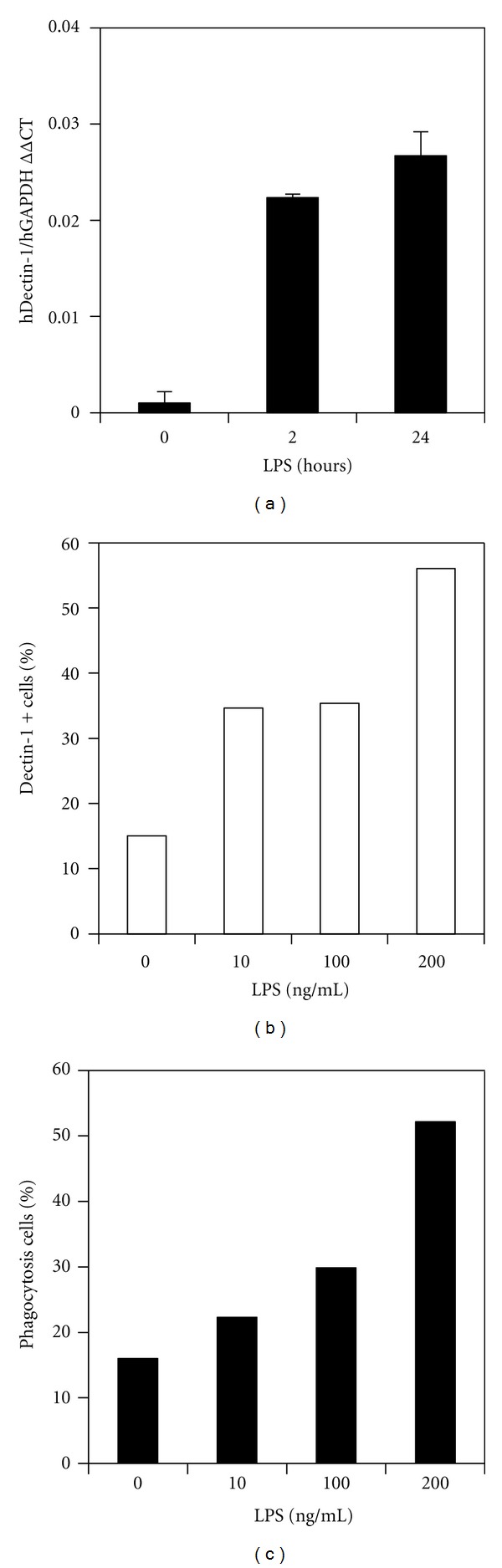
LPS-induced dectin-1 expression in freshly isolated human PBMCs. (a) The relative dectin-1 mRNA expression was detected by RT-qPCT in hPBMCs at 4 and 24 h after LPS stimulation. (b) The dectin-1 cell surface expression was shown by FACS staining. (c) Increased *Candida* phagocytosis was associated with LPS-induced dectin-1 expression by monocytes. The results were representative of 3 independent experiments.

Populations of newly isolated human PBMC contain CD14^+^ monocytes. These cells are precursors to macrophages and DCs and are able to migrate to mucosal tissues where *Candida *invasion occurs. To confirm dectin-1 expression in human PBMCs, PBMC from healthy human blood donors were stimulated for 0, 2, and 24 h with 100 ng/mL LPS. Dectin-1 mRNA expression was examined by RT-qPCR. Without LPS stimulation, freshly isolated PBMC showed low levels of dectin-1 mRNA expression. A rapid increase in dectin-1 mRNA level was detected 2 h after LPS stimulation, and increased dectin-1 expression was again evident after 24 h LPS stimulation (Figure 2(a)). Culture of PBMC in GM-CSF/IL-4 for 7 days resulted in DC maturation. We also analysed these PBMC-derived DCs for dectin-1 expression. Higher expression of dectin-1 mRNA by these cells was evident compared with PBMC. In contrast, challenge with LPS resulted in downregulation of dectin-1 expression in GM-CSF/IL-4 differentiated human PBMC (data not shown), which agrees with previous studies [30, 31]. This indicated that LPS was able to downregulate dectin-1 expression in differentiated macrophages, but it upregulated dectin-1 expression in nondifferentiated monocytes. 

After overnight culture of the isolated human PBMC in RPMI1640 full medium, approximately 15% of CD14 positive cells became dectin-1 positive monocytes, and these were readily detected using anti-dectin-1 antibody and FACS analysis. Further increases in the number of dectin-1 positive cells were evident with increasing doses of LPS stimulation (Figure 2(b)). 

To investigate the association of monocyte phagocytosis of *C. albicans* with dectin-1 expression on monocytes that were freshly isolated from peripheral blood, we stimulated the cells overnight with increasing concentrations of LPS (0 to 200 ng/mL) before addition of PI-labelled HKC for 2 h. The cell surface expression of dectin-1 was examined with an FITC-anti-dectin-1 antibody followed by FACS analysis. Cells with increased dectin-1 expression were the cells associated with HKC phagocytosis (Figure 2(c)). This result demonstrated that LPS was able to upregulate dectin-1 expression in human peripheral blood monocytes, and this in turn enhanced phagocytosis of *C. albicans.*

### 3.3. IL-23 Production by Human Monocytes following Challenge with LPS and then HKC

**Figure 3:**
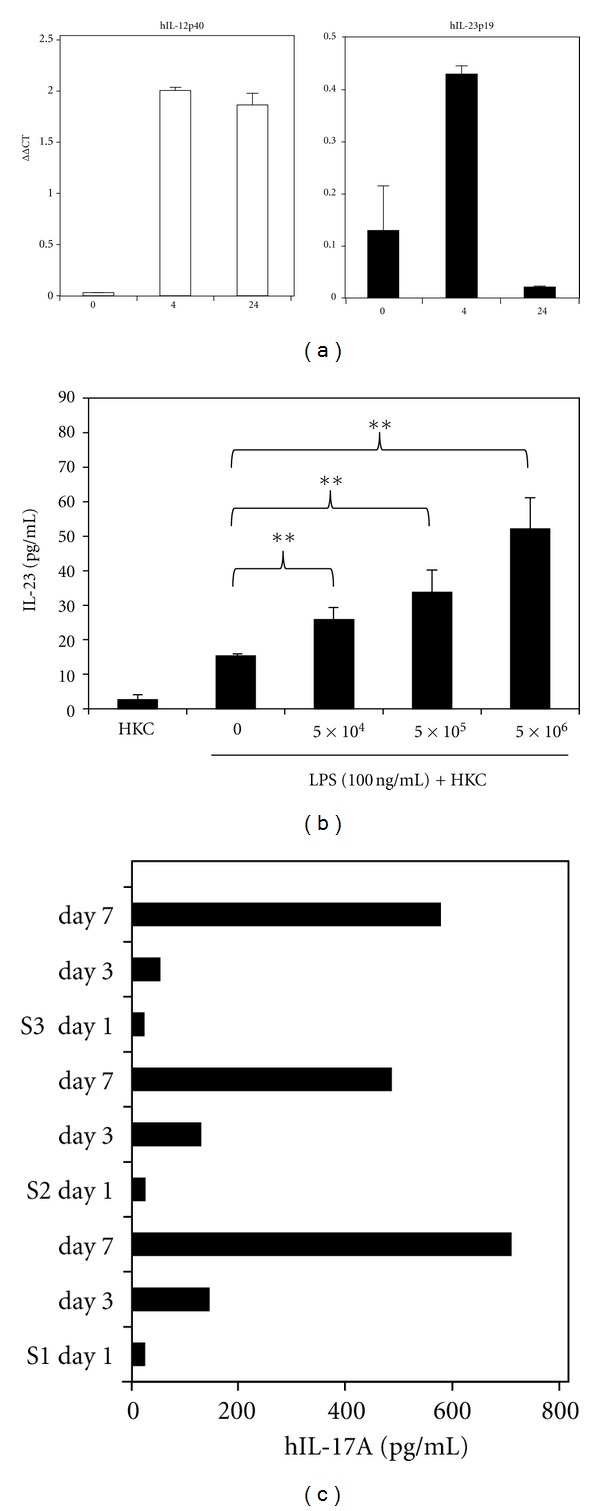
Heat-killed *Candida-*(HKC-) induced IL-23 expression in human monocytes in cell cultures containing LPS. (a) LPS pretreated human PBMC with HKC challenge and expression of mRNA for IL-12p40 and IL-23p19 subunits of IL-23. A significantly increased IL-23 production in an LPS dose-dependent manner was evident compared to cells without LPS stimulation (**P < 0.01). (b) Human monocytes cell line (THP-1) produced IL-23 in a dose-dependent manner in the cell cultures together with LPS. (c) Human PBMC produced IL-17A after days 1, 3, and 7 cultured with 3 different clinical strains of HKC.

IL-17 produced by Th17 cells plays an important role in controlling *C. albicans* infection [20, 32–35]. IL-23 is a critical cytokine for Th17 development and maintenance [32, 36–38]. IL-23 is a heterodimeric cytokine belonging to the IL-12 family of cytokines and shares one of its protein subunits (p40) with IL-12, the other subunit (IL-23p19) is unique to IL-23. Prestimulation with 10 ng/mL LPS for 2 h followed by challenge with HKC resulted in both increased IL-12p40 and IL-23p19 mRNA expression in THP-1 cells (Figure 3(a)). This confirmed that LPS potentially induced human PBMC for *Candida* recognition through IL-23 production. THP-1 cells did not produce detectable levels of IL-23 in cell culture as assessed by an IL-23 ELISA. However, increased concentrations of IL-23 were produced by THP-1 cells when they were cultured with increasing concentrations of HKC for 24 h after 100 ng/mL LPS Prestimulation for 2 h (Figure 3(b)). This further demonstrated that LPS together with *Candida *challenge stimulates human monocytes for IL-23 production. Most importantly, IL-17A production was detectable in cultures of human PBMC after 3 days with three different clinical strains of *C. albicans.* This was significantly increased again after 7 days with HKC challenge of human PBMCs (Figure 3(c)). IL-17A detected after long periods of cell culture may indicate that dectin-1 and other receptors for *Candida *recognition are only elevated after cell culture since, by FACS analysis, we could only detect limited levels of dectin-1 expression in PBMC after overnight culture without LPS (Figure 2(b)). Unfortunately, we did not detect IL-23 production in those cell cultures, which might indicate the consumption of IL-23 by Th17 cells and other cells with IL-23 receptor expression in human PBMC cultures, since PBMCs are heterogeneous cell populations. 

## 4. Discussion

Monocytes in peripheral blood are precursor cells from bone marrow. Monocytes can further differentiate into macrophages and DCs that produce cytokines, particularly those of the IL-12 family, thus bridging innate and adaptive immunity. Monocyte and macrophage/DCs are major host innate immune cells that play an important role in controlling *Candida *infection. *Candida albicans *recognition by host innate immune cells will lead to the production of cytokines, which stimulate immune cells for pathogen killing. To understand how *Candida *induces host immune cell recognition for cytokine production, we isolated clinical strains of *C. albicans* and these were heat killed before addition to a culture of human monocytes (THP-1 cell line). Unexpectedly, there was no detection of members of the IL-12 cytokine family (IL-12, IL-23, and IL-27) in the cell culture supernatant, even after 48 h. In contrast, LPS stimulated high IL-23 and IL-27 expression and in a dose-dependent manner (data not shown). These results indicated that THP-1 cells cannot recognise heat-killed *Candida* (HKC) leading to cytokine production. Dectin-1 is a C-type lectin receptor and key to *C. albicans *recognition by binding to the *β*-glucan component of the yeast cell wall. Recent studies have shown that *β*-glucan in *C. albicans* cell wall may actually be masked during the early stages of infection and then later exposed [39]. As a result, host recognition of *Candida *via *β*-glucan interaction with dectin-1 and subsequent induction of immune responses and infection control may be less prominent in early stages of *Candida* infection compared with later ones. In our *in vitro* studies, the extent of potential *β*-glucan masking was unknown, and it may have been possible that heat treatment of the *Candida* would have increased *β*-glucan exposure and thus enhanced the observed responses compared with the *in vivo* situation. Any enhancement of *β*-glucan exposure would have been consistent for all experiments. We maintain that induced dectin-1 expression by monocytes for recognition of *Candidaβ*-glucan plays a key role in controlling both mucosal and systemic *Candida *infection in humans, despite potential early masking, and importantly, dectin-1 gene mutation in patients has been associated with *Candida* infection of mucosal skin [16]. 

In this present study, we found low levels of dectin-1 expression by THP-1 cells cultured in full culture medium. LPS induced a dose-dependent increase in dectin-1 expression by these cells, as seen by both mRNA and protein levels. This was not only evident in THP-1 cells, but also in newly isolated human PBMC-derived monocytes. THP-1 cells are precursors of human macrophage/DC cell line which can be further matured by culture in cell medium containing GM-CSF. LPS stimulation therefore appears to sensitise human monocytes for *Candida *recognition. It has previously been reported that *β*-glucan together with LPS stimulated a 6-fold higher IL-10 production by freshly isolated human monocytes [29]. Stimulation by HKC together with LPS resulted in THP-1 cells producing an increased quantity of IL-23, and this was dependent on HKC cell number. This result suggests that increased dectin-1 expression by LPS subsequently resulted in higher IL-23 production following *C. albicans* challenge. IL-23 is a critical cytokine that drives and maintains Th17 responses. Th17 cells are a subpopulation of CD4^+^ T-cells which are differentiated from naïve T-cell in the tissue environment containing TGF*β*1, IL-1*β*, and IL-23 [40]. *C. albicans* can drive Th17 development with IL-17 and IFN*γ* production, but not with IL-10 [41].

Previously, other groups have shown that mouse and human matured macrophages and dendritic cells expressed higher levels of dectin-1, and reduced expression was seen with LPS stimulation [30, 31, 42, 43]. We have shown increased dectin-1 expression after culture of human PBMC with GM-CSF/IL-4 (to generate typical PBMC-derived DCs). Interestingly, LPS was found to downregulate dectin-1 expression in these cells, which agrees with previous reports. However, monocytes without full maturation expressed considerably lower levels of dectin-1, and a significantly increased dectin-1 expression was then observed after LPS stimulation. The different levels of dectin-1 mRNA expression for human PBMC and THP-1 cells (as shown by hDectin-1/hGAPDH ΔΔCT) may be explained by a relatively lower cell number in PBMCs. FACS analysis showed that about 10% of human PMBCs were CD14^+^ monocytes. Amongst these cells, 10% CD14^+^ monocytes expressed dectin-1 after overnight culture with LPS stimulation, whilst around 55% cells became dectin-1 positive after 200 ng/mL LPS stimulation. 

In the present study, PBMC cultured with HKC-induced expression of mRNA for both IL-23 subunits. Although IL-23p19 mRNA was rapidly and transiently expressed, IL-12p40 was maintained at a higher level after 24 h culture. IL-23 was not detectable by ELISA in human PBMC cell culture supernatant, and this might indicate its consumption by T-cells within the culture. IL-17 production was detected in the cell culture following HKC stimulation, but not in controls at days 3 and 7. This may indicate that the Th17 recalled response, which requires days to become fully functional. In this study, three different clinical isolates of *Candida* were added to the cell cultures, and no significant difference in terms of IL-17 production was evident between these strains. 

Apart from stimulation of cytokine IL-23 production in human monocytes by HKC, we also found that increased expression of dectin-1 in human monocytes was associated with *Candida *phagocytosis. It has recently been reported that *β*-glucan particles bound to dectin-1 in macrophages form a phagocytic synapse that initiates cell phagocytosis and cytokine production [14]. Our results further confirm that dectin-1 induced by LPS has a critical role in *Candida *control. 

Within the body, monocytes that migrate from the bone marrow and circulate in the blood do not require dectin-1 expression, and this was evident in our study with newly isolated human PBMC. However, *Candida* colonisation at mucosal surfaces will result in the tissue producing GM-CSF and IL-4 plus various chemokines for recruitment and maturation of monocytes to macrophages and dendritic cells. The function of these matured phagocytotic cells will be the recognition, phagocytosis, and killing of the infecting *Candida*. Consequently, GM-CSF-induced dectin-1 expression in macrophages and DCs is essential to prepare these cells for these purposes. This too was evident in our study, following incubation of the human PBMC with GM-CSF and IL-4.

It was somewhat surprising that LPS was found to downregulate dectin-1 expression in matured DCs and macrophages. Such downregulation is normally associated with high inflammatory cytokine production such as TNF*α*, IFN*γ*, and IL-12p70. At early stages of infection, it could be that LPS alters human innate immune cells (monocytes) by stimulating dectin-1 expression leading to *C. albicans* recognition. However, in later stages and after these monocytes have matured into macrophages and DCs with sufficiently high dectin-1 cell surface expression, LPS may then trigger the cell signal to suppress dectin-1 and stimulate proinflammatory cytokine production to enforce inflammatory responses. 

Understanding the phenotypic changes of macrophages that occur due to LPS presence is key to elucidating the mechanisms of wound healing and infection resolution. In this study, we have shown that LPS is able to induce dectin-1 expression in human monocytes, and this results in IL-23 production with enhanced *Candida* phagocytosis. LPS may thus alter innate immune cell function for *Candida *recognition and affect early stages of *Candida* infection.

## References

[1] Haas A, Zimmermann K, Graw F (2011). Systemic antibody responses to gut commensal bacteria during chronic HIV-1 infection. *Gut*.

[2] Paquet P, Piérard-Franchimont C, Piérard GE, Quatresooz P (2008). Skin fungal biocontamination and the skin hydrogel pad test. *Archives of Dermatological Research*.

[3] Wei XQ, Rogers H, Lewis MAO, Williams DW (2011). The role of the IL-12 cytokine family in directing T-cell responses in oral candidosis. *Clinical and Developmental Immunology*.

[4] Vellappally S, Fiala Z, Smejkalová J, Jacob V, Somanathan R (2007). Smoking related systemic and oral diseases. *Acta Medica*.

[5] Dawes C (2008). Salivary flow patterns and the health of hard and soft oral tissues. *Journal of the American Dental Association*.

[6] Salerno C, Pascale M, Contaldo M (2011). Candida-associated denture stomatitis. *Medicina Oral, Patologia Oral y Cirugia Bucal*.

[7] Alajbeg I, Vucićević-Boras V (2002). Burning mouth syndrome—etiologic, diagnostic and therapeutic considerations. *Liječnički Vjesnik*.

[8] Gonsalves WC, Chi AC, Neville BW (2007). Common oral lesions: part I. Superficial mucosal lesions. *American Family Physician*.

[9] Junqueira JC (2012). Models hosts for the study of oral candidiasis. *Advances in Experimental Medicine and Biology*.

[10] Strasser D, Neumann K, Bergmann H (2012). Syk kinase-coupled C-type lectin receptors engage protein kinase C-sigma to elicit Card9 adaptor-mediated innate immunity. *Immunity*.

[11] Iliev ID, Funari VA, Taylor KD (2012). Interactions between commensal fungi and the C-type lectin receptor dectin-1 influence colitis. *Science*.

[12] Brown GD, Gordon S (2001). Immune recognition. A new receptor for *β*-glucans. *Nature*.

[13] Taylor PR, Tsoni SV, Willment JA (2007). Dectin-1 is required for *β*-glucan recognition and control of fungal infection. *Nature Immunology*.

[14] Goodridge HS, Reyes CN, Becker CA (2011). Activation of the innate immune receptor Dectin-1 upon formation of a ‘Phagocytic synapse’. *Nature*.

[15] Gringhuis SI, Kaptein TM, Wevers BA, Theelen B, Boekhout T (2012). Dectin-1 is an extracellular pathogen sensor for the induction and processing of IL-1*β* via a noncanonical caspase-8 inflammasome. *Nature Immunology*.

[16] Ferwerda B, Ferwerda G, Plantinga TS (2009). Human dectin-1 deficiency and mucocutaneous fungal infections. *New England Journal of Medicine*.

[17] LeibundGut-Landmann S, Groß O, Robinson MJ (2007). Syk- and CARD9-dependent coupling of innate immunity to the induction of T helper cells that produce interleukin 17. *Nature Immunology*.

[18] Rogers NC, Slack EC, Edwards AD (2005). Syk-dependent cytokine induction by dectin-1 reveals a novel pattern recognition pathway for C type lectins. *Immunity*.

[19] Gringhuis SI, den Dunnen J, Litjens M (2009). Dectin-1 directs T helper cell differentiation by controlling noncanonical NF-*κ*B activation through Raf-1 and Syk. *Nature Immunology*.

[20] Conti HR, Shen F, Nayyar N (2009). Th17 cells and IL-17 receptor signaling are essential for mucosal host defense against oral candidiasis. *Journal of Experimental Medicine*.

[21] Wakabayashi H, Takakura N, Teraguchi S, Tamura Y (2003). Lactoferrin feeding augments peritoneal macrophage activities in mice intraperitoneally injected with inactivated. *Microbiology and Immunology*.

[22] Rajkovic I, Dragicevic A, Vasilijic S (2011). Differences in T-helper polarizing capability between human monocyte-derived dendritic cells and monocyte-derived Langerhans'-like cells. *Immunology*.

[23] Swanson JA (2008). Shaping cups into phagosomes and macropinosomes. *Nature Reviews Molecular Cell Biology*.

[24] Kinchen JM, Doukoumetzidis K, Almendinger J (2008). A pathway for phagosome maturation during engulfment of apoptotic cells. *Nature Cell Biology*.

[25] Underhill DM, Ozinsky A (2002). Phagocytosis of microbes: complexity in action. *Annual Review of Immunology*.

[26] Goodridge HS, Underhill DM (2008). Fungal recognition by TLR2 and dectin-1. *Handbook of Experimental Pharmacology*.

[27] Káposzta R, Maródi L, Hollinshead M, Gordon S, Da Silva RP (1999). Rapid recruitment of late endosomes and lysosomes in mouse macrophages ingesting *Candida albicans*. *Journal of Cell Science*.

[28] Malic S, Hill KE, Ralphs JR (2007). Characterization of *Candida albicans* infection of an in vitro oral epithelial model using confocal laser scanning microscopy. *Oral Microbiology and Immunology*.

[29] Chen L, Wei XQ, Evans B, Jiang W, Aeschlimann D (2008). IL-23 promotes osteoclast formation by up-regulation of receptor activator of NF-B (RANK) expression in myeloid precursor cells. *European Journal of Immunology*.

[30] Reid DM, Montoya M, Taylor PR (2004). Expression of the *β*-glucan receptor, Dectin-1, on murine leukocytes in situ correlates with its function in pathogen recognition and reveals potential roles in leukocyte interactions. *Journal of Leukocyte Biology*.

[31] Bonfim CV, Mamoni RL, Lima Blotta MHS (2009). TLR-2, TLR-4 and dectin-1 expression in human monocytes and neutrophils stimulated by *Paracoccidioides brasiliensis*. *Medical Mycology*.

[32] Navarathna DHMLP, Nickerson KW, Duhamel GE, Jerrels TR, Petro TM (2007). Exogenous farnesol interferes with the normal progression of cytokine expression during candidiasis in a mouse model. *Infection and Immunity*.

[33] Raška M, Běláková J, Křupka M, Weigl E (2007). Candidiasis—do we need to fight or to tolerate the *Candida* fungus?. *Folia Microbiologica*.

[34] Dimitrova P, Yordanov M, Danova S, Ivanovska N (2008). Enhanced resistance against systemic *Candida albicans* infection in mice treated with *C. albicans* DNA. *FEMS Immunology and Medical Microbiology*.

[35] Saunus JM, Wagner SA, Matias MA, Hu Y, Zaini ZM, Farah CS (2010). Early activation of the interleukin-23-17 axis in a murine model of oropharyngeal candidiasis. *Molecular Oral Microbiology*.

[36] Rivas V, Rogers TJ (1983). Studies on the cellular nature of *Candida albicans*-induced suppression. *Journal of Immunology*.

[37] Sachdeva N, Weinstein JE, Ashman M (2010). Poor lymphoproliferative responses with low proportion of gag-specific CD8 TEMRA cells in HIV-1-infected patients showing immunological and virological discordance despite prolonged suppression of plasma viremia. *Viral Immunology*.

[38] Kalo-Klein A, Witkin SS (1990). Prostaglandin E2 enhances and gamma interferon inhibits germ tube formation in *Candida albicans*. *Infection and Immunity*.

[39] Wheeler RT, Kombe D, Agarwala SD, Fink GR (2008). Dynamic, morphotype-specific *Candida albicansβ*-glucan exposure during infection and drug treatment. *PLoS Pathogens*.

[40] Perfect JR, Wright KA (1994). Amphotericin B lipid complex in the treatment of experimental cryptococcal meningitis and disseminated candidosis. *Journal of Antimicrobial Chemotherapy*.

[41] Reitan LJ, Closs O, Belehu A (1982). In vitro lymphocyte stimulation in patients with lepromatous and borderline tuberculoid leprosy. The effect of dapsone treatment on the response to Mycobacterium leprae antigens, tuberculin purified protein derivative and non-mycobacterial stimulants. *International Journal of Leprosy*.

[42] Willment JA, Marshall AS, Reid DM (2005). The human *β*-glucan receptor is widely expressed and functionally equivalent to murine Dectin-1 on primary cells. *European Journal of Immunology*.

[43] Willment JA, Lin H-H, Reid DM, Wong SYC, Brown GD (2003). Dectin-1 expression and function are enhanced on alternatively activated and GM-CSF-treated macrophages and are negatively regulated by IL-10, dexamethasone, and lipopolysaccharide. *Journal of Immunology*.

